# LABOR MARKET PROSPECTS AND FINANCIAL AND HEALTH OUTCOMES OF ADULT CHILDREN CAREGIVERS AFTER THEIR PARENTS’ DEATH

**DOI:** 10.1093/geroni/igae098.3095

**Published:** 2024-12-31

**Authors:** Stipica Mudrazija, Katherine Ornstein, Fernando Hernandez

**Affiliations:** University of Washington, Seattle, Washington, United States; Johns Hopkins University, Baltimore, Maryland, United States; Urban Institute, Washington, District of Columbia, United States

## Abstract

As the population ages, an increasing number of adult children provide unpaid care to their parents who require help with health or function. While research and policy attention has focused on improving the caregiving experience, including workplace leave practices, little is known about the outcomes and challenges of family members once their caregiving role ends post-death. The context of caregiving, including factors such as the age of deceased parent, duration of care, level of (family) support, and caregiver gender, among others, may all impact the labor force trajectory and other adult children caregivers’ outcomes following their parents’ death. For this analysis, we use data from the 1998-2020 waves of the Health and Retirement Study, focusing on labor market trajectories, financial wellbeing and health outcomes of former caregivers ages 53-61 at the time of their parents’ death. Our preliminary results suggest that within two years of their parents’ death, adult children caregivers remain 6 percentage points less likely to work than those whose parents have not passed away. This suggests that former caregivers continue to be less likely to work than non-caregivers following the death of their parents. Additional analyses will focus on examining longer term labor force trajectory of former family caregivers as well as other outcomes of interest, including their financial wellbeing and physical and mental health status.

